# An Unusual *Salmonella* Enteritidis Strain Carrying a Modified Virulence Plasmid Lacking the *prot6e* Gene Represents a Geographically Widely Distributed Lineage

**DOI:** 10.3389/fmicb.2020.01322

**Published:** 2020-06-17

**Authors:** Susan Nadin-Davis, Louise Pope, John Chmara, Marc-Olivier Duceppe, Teresa Burke, John Devenish, Olga Andrievskaia, Ray Allain, Dele Ogunremi

**Affiliations:** Ottawa Laboratory Fallowfield, Canadian Food Inspection Agency, Ottawa, ON, Canada

**Keywords:** *Salmonella* Enteritidis lineage, virulence plasmid, MLVA, whole genome sequencing, PFGE, SNP-based typing, phage type

## Abstract

This study identifies a strain of *Salmonella enterica* subspecies *enterica* serovar Enteritidis that harbors a highly unusual virulence plasmid. During the characterisation of a group of *S.* Enteritidis isolates, 10 isolates recovered from Canadian duck production facilities, of which seven were phage type 9b and three were closely related atypical phage types, failed detection by a PCR targeting the *prot6e* gene, a marker located on the virulence plasmid often employed for identification of this serovar. Comparison to *prot6e*+ isolates by several standard genetic typing tools, further revealed their distinctive genomic makeup. Both short read and long read whole genome sequencing were completed on six of these isolates. In addition to loss of the *prot6e* gene, the virulence plasmid of each isolate was found to be exceptionally large (86.5 Kb) due to a 28 Kb insertion of *S*. Typhimurium plasmid sequence that encodes multiple genes of the *incF* operon. Interrogation of the chromosome sequence data of these isolates using a SNP-based typing tool and MLST both indicated their close genetic relatedness. One additional isolate carrying this plasmid was identified in an in-house collection of *S.* Enteritidis isolates. Finally, the identification of this unusual plasmid sequence in additional isolates submitted to public repositories of *Salmonella* sequence data was explored. All these analyses indicated that a very distinctive but rarely reported strain of *S*. Enteritidis was widely distributed across North America and the United Kingdom with one additional report involving a case from Brazil. With increased use of genetic methods for *Salmonella* identification, the loss of the *prot6e* sequence may confound correct identification of this serovar while also potentially altering the mode of transmission to humans given the gene’s role in facilitating propagation of this bacterium in eggs. Accordingly, this strain may present certain challenges with respect to public health investigations. Our studies also suggest this strain is often associated with duck hosts thereby providing a possible mechanism by which this strain has spread over an extensive geographical area.

## Introduction

In most countries, including developed nations such as Canada, the United States and those of Europe, *Salmonella* Enteritidis is recognized as the major *Salmonella* serotype contributing to human gastroenteritis. Poultry products are a significant source of foodborne contamination by this pathogen (Chai et al., 2012; Nesbitt et al., 2012; Taylor et al., 2012; Lane et al., 2014; Middleton et al., 2014) a fact that has resulted in major food recalls, including one that involved over 500 million eggs in the United States in 2010 (Kuehn, 2010). In Canada *S.* Enteritidis, *S.* Typhimurium and *S*. Heidelberg have been recognized as the three most frequently reported *Salmonella* serotypes for several years, and together they comprised 59% of all laboratory–confirmed cases of human salmonellosis in 2016 (PHAC, 2018). Environmental testing for *Salmonella* in poultry production facilities is one tool employed in efforts to limit contamination of food by these organisms. To this end traditional culture methods for *Salmonella* detection are now being augmented by more rapid, sensitive and specific molecular genetic based methods.

The use of a panel of qPCRs, targeting markers of various levels of specificity, has recently been evaluated for the purpose of rapid detection of *Salmonella*, and specifically *S*. Enteritidis, in culture broths generated from environmental swabs (Nadin-Davis et al., 2019). This panel targeted the *invA* gene to detect all *Salmonella*, the *sefA* gene for presumptive serogroup D identification and two loci for more specific detection of *S*. Enteritidis namely, the chromosomal locus *sdf1* and the *prot6e* gene. These analyses revealed that a sub-group of isolates, all confirmed by serotyping as *S.* Enteritidis and having either atypical or 9b phage types, were positive for the chromosomal *sdfI* marker but negative for the *prot6e* gene located on the *S*. Enteritidis virulence plasmid and which is believed to contribute to the ability of *S*. Enteritidis to survive in and be transmitted via eggs (Clavijo et al., 2006). Given that the *prot6e* gene is frequently used for *S*. Enteritidis detection (Malorny et al., 2007; Chiang et al., 2018) it was felt that further characterisation of these *prot6e*-negative isolates was warranted. It was notable that all these *prot6e-* isolates originated from facilities identified as duck-producing facilities while all the *prot6e+* isolates came from chicken or more general poultry-producing facilities. Due to the clonal nature of *S*. Enteritidis, several subtyping schemes have been explored in attempts to improve lineage discrimination and thereby improve trace back capability following food contamination events. Pulsed field gel electrophoresis (PFGE), the gold standard bacterial typing technique widely applied to *Salmonella* in food outbreak investigations until very recently (Ribot et al., 2006; Peters et al., 2007) had limited capability to differentiate *S*. Enteritidis isolates. An alternative bacterial subtyping technique, Multiple-Locus Variable number tandem repeat Analysis (MLVA), has proven useful (Nadon et al., 2013) and a standardized protocol has been employed for epidemiological investigations of *S*. Enteritidis worldwide to supplement traditional PFGE subtyping (Bertrand et al., 2015). More recently *in silico* analyses of whole genome sequencing (WGS) data have increasingly been adopted as the tools of choice for both serovar identification (Yoshida et al., 2016) and highly sensitive isolate subtyping in support of salmonellosis outbreak investigations (Ogunremi et al., 2014b). Using such methods for a comprehensive characterisation of these *prot6e-*negative *S*. Enteritidis isolates has revealed the existence of a highly distinct lineage having unique features in both chromosomal and plasmid sequences. The broader circulation of this lineage has been evaluated using multiple sequence databases and the public health implications of these observations are considered.

## Materials and Methods

### Collection of *Salmonella* Isolates

All 63 isolates (Supplementary Table S1) included in the laboratory studies were recovered from environmental samples taken in poultry production facilities and submitted to the Animal Health Microbiology Diagnostic Unit of the Canadian Food Inspection Agency for *Salmonella* detection. Culture of these environmental samples, performed as described previously (Nadin-Davis et al., 2019) yielded individual colonies which were cultured on tryptic soy agar (TSA) with or without 5% sheep blood to assess purity. All isolates were identified to the genus *Salmonella* using Vitek 2 (bioMérieux, Durham, NC, United States) according to the manufacturer’s instructions and the serovar, Enteritidis, of isolates along with the phage type, was confirmed by serological and phage typing analysis performed by the World Health Organisation Reference Centre for *Salmonella* located in a Public Health Agency of Canada facility in Guelph, Ontario.

### DNA Extraction and qPCR

Pure isolates of *S.* Enteritidis were grown on TSA plates and incubated at 36°C for 18–22 h. Cells were scraped off the agar surface and resuspended in PBS prior to total DNA extraction using a Wizard genomic DNA isolation kit as per the supplier’s directions (Promega, Madison, WI, United States). Purified DNA solutions were quantified spectroscopically using a Nanovue instrument (GE Biosciences) and stored at −20°C. Isolates were scored for the presence of the *sdf1* locus and the *prot6e* gene by qPCR performed as described previously (Nadin-Davis et al., 2019).

### Pulse Field Gel Electrophoresis

PFGE was performed using the PulseNet Canada method “General Pulsed Field Gel Electrophoresis for *Escherichia coli*, *Salmonella*, and *Shigella*” (PulseNetInternational, 2013) with the following modifications: plug slices were separately digested with 120 units *XbaI* and 40 units *BlnI* with extended digestion times of a minimum of 3 h and electrophoresis run time of approximately 20 h on the CHEF Mapper (Bio-Rad Laboratories, Mississauga, ON, Canada). Data were analyzed using the software BioNumerics v6.01 (Applied Maths, Sint-Martens-Latem, Belgium), and PFGE patterns were assigned by PulseNet Canada (National Microbiology Laboratory, Winnipeg, Canada).

### Multiple-Locus Variable-Number Tandem-Repeat Analysis (MLVA)

Each of the 63 cultured *S*. Enteritidis isolates was used to prepare a crude cell lysate by heating 1–3 colonies in 100 μl molecular grade water at 100°C for 10 min. These crude cell lysates were used as DNA templates in MLVA typing procedures performed according to the standardized PulseNet protocol (PulseNetInternational, 2011). Amplification was performed on ABI-9700 thermal cyclers (Applied Biosystems, CA) with cycling parameters of 95°C for 5 min, followed by 35 cycles of 94°C for 20 s, 65°C for 20 s and 72°C for 20 s, and a hold at 72°C for 5 min. The variable-number tandem-repeat (VNTR) PCR products were analyzed by capillary electrophoresis on an ABI-3130xl Genetic Analyzer (Applied Biosystems). In cases when PCR products were absent for a VNTR locus, a confirmatory singleplex PCR was performed using the appropriate VNTR primer set. VNTR fragment lengths were calculated using the GeneMapper v4.1 software (Life Technologies), and VNTR allele numbers were assigned using BioNumerics v6.01 as per an algorithm provided by PulseNet Canada. Final MLVA types were presented as allele number strings VNTR 1-VNTR 2-VNTR 3-VNTR 5-VNTR 6-VNTR 8-VNTR 9 indicating the number of tandem repeats within the corresponding VNTR loci. An absent VNTR locus was scored as an allele (VNTR allele number = nd) reflecting either loss of the locus or a polymorphic primer binding site of the given locus. The MLVA dendrogram was generated by BioNumerics v6.01 using the categorical coefficient and Unweighted Pair Group Method with Arithmetic Mean (UPGMA) method; MLVA clusters were defined by a cutoff value of 57% similarity.

### Whole Genome Sequencing

Whole genome sequencing (WGS) was initially performed on seven *prot6e- S.* Enteritidis isolates using two different short read platforms; results for six isolates are summarized in Table 1. For sequencing by Illumina technology libraries were constructed using Nextera XT kits and run on a MiSeq instrument with either a 500 or 600 cycle reagent kit. For Ion Torrent PGM sequencing, DNA libraries were prepared by enzymatic fragmentation, adaptor ligation and size selection of DNA according to manufacturer’s instructions (Thermo Fisher, Burlington, ON, Canada) and converted to sequencing templates displayed on Ion sphere particles by means of an emulsion PCR (Ion One-Touch ES^TM^). Ion particles enriched with templates were loaded on the Ion Torrent 318 chip and the DNA sequenced on the PGM over 7 h as per the manufacturer’s directions. Additional long read sequencing was performed on these six isolates using Nanopore MinION technology to confirm the plasmid assemblies and assist with assembly of whole chromosome data. Libraries were generated using a rapid sequencing kit with barcoding as per manufacturer’s instructions and run on a R9.4 MinION flow cell for 24 h (Oxford Nanopore).

**TABLE 1:** Sequence summary of the virulence plasmid for six unusual *S.* Enteritidis isolates and comparison with reference strains.

Isolate designation	*Salmonella* Serovar	Plasmid ID	Sequence platforms^1^	Plasmid size (bp)	% GC	# Non-conserved positions^2^	# SNPs^2^	# Indels^2^	# Coding features	*incF* conjugative transfer system^3^	*traD* # of 9 bp repeats^4^	NCBI Accession #
06D1274 20-15	Enteritidis	p06D1274	PGM/Min	86,504	52.6	82	8	74	107	33^5^	7	NZ_VYYH01000002.1
08D015 15-1	Enteritidis	p08D015	Ill/Min	86,472	52.6	55	3	52	107	32	6	NZ_VYYG01000005.1
08OTH027 7-4	Enteritidis	p08OTH027	PGM/Min	86,562	52.6	143	7	136	108	32	14	NZ_VYYF01000002.1
11OTH031 8-1	Enteritidis	p11OTH031	Ill/Min	86,450	52.6	0	0	0	107	32	2	NZ_VYYE01000003.1
12OTH012 9-1	Enteritidis	p12OTH012	PGM/Ill/Min	86,537	52.6	234	71	163	104	32	14	NZ_VYYD01000004.1
14OTH007 19-16	Enteritidis	p14OTH007	Ill/Min	86,650	52.6	215	9	206	105	32	23	NZ_VYYC01000002.1
**Reference strains**												
LT2	Typhimurium	pSLT	–	93,933	53.1	n/d			119	31	11	AE006471.2
P125109	Enteritidis	pSEN	–	59,372	51.9	n/d			78	14	Not present	HG970000

*^1^Ill, Illumina; Min, Nanopore MinION; PGM, Ion Torrent Personal Genome Machine. ^2^Using p11OTH031 as reference. ^3^All six Enteritidis isolates encode a truncated *traG*, no *traS* and an additional *traJ-5* gene. ^4^Repeat sequence CAGCCGCAA. ^5^Has an additional predicted *tral* gene.*

### Sequence Assembly

Initially short read sequence data were used for plasmid sequence assembly after removal of chromosomal DNA sequences using the reference *S.* Enteritidis strain P125109 (NCBI accession no. AM933172.1) with the Ngen program of the Lasergene v12 software (DNASTAR, Madison, WI, United States). Gaps in the resulting assemblies were filled by Sanger sequencing of PCR products bridging these gaps. The later acquisition of long read sequence data using MinION technology permitted confirmation and refinement of these plasmid sequences in addition to the generation of whole chromosome assemblies. The combined sequence data from all platforms (Illumina paired-end, Ion Torrent, and MinION reads) were assembled using Unicycler v0.4.8-beta (Wick et al., 2017) which employs the SPAdes tool for *de novo* assembly of the short read data and then uses the long reads to bridge gaps in these assemblies^1^. The bridged assemblies then underwent multiple rounds of short-read polishing to generate the final sequences described in this report. All plasmid sequences presented here were oriented such that the *repB* open reading frame started at position 1 in the positive orientation. Differences between complete whole genome assemblies of the six unusual *S.* Enteritidis samples were identified through the mapping of short Illumina or Ion Torrent sequence reads to the 11OTH031 hybrid assembly using Bowtie2 v2.3.4.3, and subsequently merging and calling variants from the output files with the BCFtools module of SAMtools v1.9 (Li et al., 2009). To confirm the MLVA subtyping analysis these genome sequences were evaluated for the presence/absence of the MLVA locus VNTR 3 primers using an in-house *in silico* PCR pipeline^2^ in which up to 3 mismatches were permitted per primer.

The hybrid whole genome sequence assemblies have been deposited into the NCBI database and have the following accession numbers: 2006D1274_20-15, GCA_008728015.1; 2008D015_15-1, GCA_008727965.1; 2008OTH027_7-4, GCA_008727955.1; 2011OTH031_8-1, GCA_008727985.1; 2012OTH012_9-1, GCA_008727855.1; 2014OTH007_19-16, GCA_008727915.1 Note that in the following narrative the designations have been shortened to include just a two digit year identifier. Details of the assembled plasmid sequences are provided in Table 1.

### *S.* Enteritidis SNP Subtyping

Nucleotides at the 60 chromosomal positions used for SNP typing as described previously (Ogunremi et al., 2014b) were recovered from the assembled chromosomal sequences by mapping the raw Illumina reads to the genome of the reference *S.* Enteritidis strain P125109. Two positions not resolved from these data and interrogated using the raw reads were found not to be present and were scored as gaps. Using the tools available in the MEGA X software suite (Kumar et al., 2018) these data were aligned with SNP reads for other *S*. Enteritidis isolates representative of the 12 clades identified previously (Ogunremi et al., 2014b). This alignment was then used to generate a neighbor-joining tree using the Maximum Composite Likelihood method and 2000 bootstrap replicates with complete removal of all positions containing gaps. The alignment was also used to generate SNP differences among the isolates by pairwise comparison (CLC Genomics Workbench v 12, Qiagen, Hilden, Germany).

### Plasmid Sequence Comparisons and Annotations

Using the sequence of the virulence plasmid from one of the isolates, namely p11OTH031 as reference, all assembled plasmid sequences were analyzed by a pairwise alignment implemented in MEGA v7 software (Kumar et al., 2016) to identify SNPs and non-conserved positions. These six assemblies were initially annotated using Prokka v1.13 (Seemann, 2014) with further refinement using an in-house script^3^ that uses a clustering algorithm to improve predicted annotation descriptions; this second step markedly reduced the number of hypothetical open reading frames (ORFs) identified. The few annotation differences thus detected between the six plasmids were further assessed for their accuracy as follows. Sample reads were aligned to the plasmid sequence p11OTH031 using Bowtie2 v2.3.4.3 (Langmead and Salzberg, 2012), and the resultant files were filtered and formatted using the BCFtools module of SAMtools v1.9 (Li et al., 2009)^4^. Differences were filtered based on read-mapping and quality threshold values of 20 for all samples so as to remove poor quality data. Alignments of these plasmid sequences with those for the plasmids of two reference strains, S. Enteritidis P125109 (pSEN) and *S.* Typhimurium LT2 (pSLT), were illustrated graphically using the BRIG v0.95 (Alikhan et al., 2011) software program.

### Comparison of *S.* Enteritidis Plasmid Sequences With CFIA In-House and Enterobase Sequence Archives

A collection of hundreds of *Salmonella* isolates, including 240 *S*. Enteritidis samples, recovered from across Canada as part of the CFIA’s food safety program and sequenced on the MiSeq platform using the methods described above, were interrogated to determine if additional isolates harboring this unusual plasmid could be identified. Raw sequence reads were interrogated with the sequence of the reference plasmid p06D1274 using the nucmer module of MUMmer v3.23 (Marçais et al., 2018). To ensure identification of *S.* Enteritidis isolates only, pairwise comparisons between the reference plasmid and sample reads were catalogued using the DNAdiff script contained within MUMmer with parsing of the data by adjusting the threshold level of coverage until removal of *S*. Typhimurium samples was achieved.

Using an *in silico* multi-locus sequence typing (MLST) method based on the Achtman seven gene scheme for *Salmonella* implemented through the Enterobase website^5^ the six isolates fully characterized in this study were all typed as ST-814. All 128 available ST-814 assemblies were downloaded from the Enterobase database and interrogated using the nucmer and DNAdiff modules of MUMmer v3.23 (Marçais et al., 2018) and the p06D1274 reference sequence as described above. Of the 54 assemblies that were returned by this analysis, only 45 had available short read archive (SRA) entries in NCBI. Using these SRA entries, samples were analyzed using SNVPhyl v1.0.1 (Petkau et al., 2017) for single nucleotide variants in these whole genome sequences. Single nucleotide variants present in each isolate as identified using the SNVphyl software were concatenated and saved as a fasta file. MEGA v7 software was used to first determine the best fitting nucleotide substitution model for the resulting data and then to predict the most likely phylogeny using the Maximum Likelihood (ML) approach. This fasta file was also used to compute SNP differences and percent identity (CLC Genomics Workbench v 12) by pairwise comparison of members of clades identified by the ML phylogeny. To evaluate the two main clades identified in the ML tree, their distribution of SNP differences was tested for normality (Kolmogrov–Sminrnov test, Clade I KS distance = 0.12, *p* < 0.05; Clade II, KS distance = 0.15, *p* < 0.05), and significant differences between the two groups tested by the non-parametric Mann–Whitney test at *p* < 0.05 (GraphPad Prism 8, San Diego, CA, United States).

## Results

### Identification of *S*. Enteritidis Isolates With Unusual Typing Profiles

**FIGURE 1:**
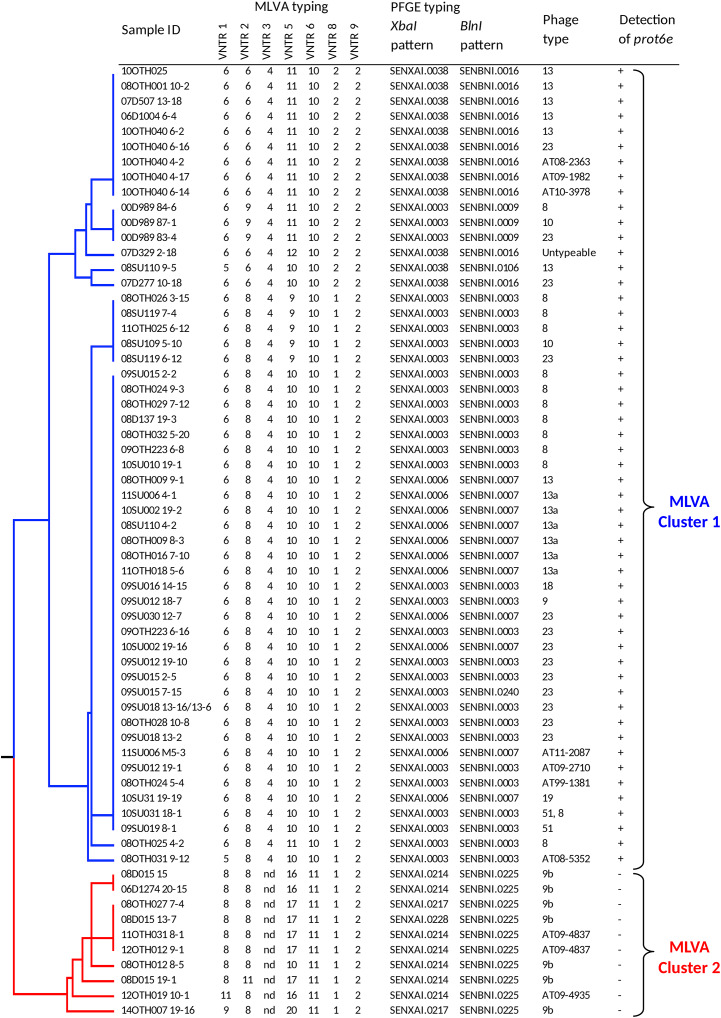
Dendrogram representing genetic relationships among 63 *S*. Enteritidis based on MLVA profiles. The table to the right of the dendrogram shows isolate IDs, the allelic pattern for each MLVA profile, PFGE and phage types, and presence of the *prot6e* gene. Division of the samples into two very distinct MLVA clusters, 1 (blue) and 2 (red), is indicated to the right.

To provide context for this study, a total of 63 *S*. Enteritidis isolates, recovered from environmental samples that had been collected at multiple poultry facilities across Canada between 2000 and 2014 as part of a CFIA surveillance program, were characterized by several internationally recognized and standardized *S.* Enteritidis subtyping methods including phage type (PT) determination, PFGE and MLVA (Supplementary Table S1). These data are presented in Figure 1 together with the outcome of PCR testing for the presence of the *prot6e* marker.

MLVA subtyping generated 15 types that clustered into two major groups on the UPGMA dendrogram. Cluster 1 (*n* = 53) included the most common MLVA types 6-8-4-10-10-1-2 (*n* = 31) and 6-6-4-11-10-2-2 (*n* = 9), and seven closely related types represented by 13 isolates. This cluster of isolates was characterized by six PFGE types, the most common being SENXAI.0003 SENBNI.0003 (49.1%), SENXAI.0006 SENBNI.0007 (22.6%), and SENXAI.0038 SENBNI.0016 (20.8%). These 53 isolates were represented by 9 phage types, among which the PT23 was the most dominant (*n* = 13), followed by PT8 (*n* = 12) and PT13 (*n* = 7); seven isolates had atypical phage types and one was untypeable. All *S.* Enteritidis isolates belonging to MLVA Cluster 1 were positive for the *prot6e* marker (*p* < 0.0001).

The MLVA Cluster 2 (*n* = 10) included the MLVA type 8-8-nd-17-11-1-2 (*n* = 5) and five closely related types with the distinguishing absence of the VNTR 3 locus (position 2073266 – 2073463 in the chromosome of *S.* Enteritidis strain P125109). These samples were characterized by the PFGE profile SENXAI.0214 SENBNI.0225 and two other profiles having minor differences in the *XbaI* pattern only. Seven had the phage type PT9b while the remaining three were represented by two closely related atypical phage types. Notably all MLVA Cluster 2 isolates, which were recovered exclusively from duck hatcheries (Supplementary Table S1), were associated with negative *prot6e* qPCR results (*p* < 0.0001) while all 63 *S.* Enteritidis isolates were positive for the *sdf1* marker.

### WGS and Plasmid Analysis

To explain the failure of the *prot6e* qPCR assay for *S*. Enteritidis isolates of MLVA Cluster 2, seven isolates were initially selected for short read WGS using one of two platforms in each case. Subsequently just six of these isolates were also subjected to long-read sequencing since the seventh isolate (12OTH019 10-1) was no longer available for Nanopore sequencing (Table 1). The use of multiple sequencing methods, in which both short and long read chemistries were employed, allowed for highly robust plasmid sequence assemblies. While the plasmid sequence predicted for isolate 12OTH019 10-1 was based upon short read data only, it was very similar to those of the other isolates indicating that in this study even short read data was assembled quite accurately. In each case a single, highly conserved large virulence plasmid of ∼86.5 kb was identified (Table 1).

**FIGURE 2:**
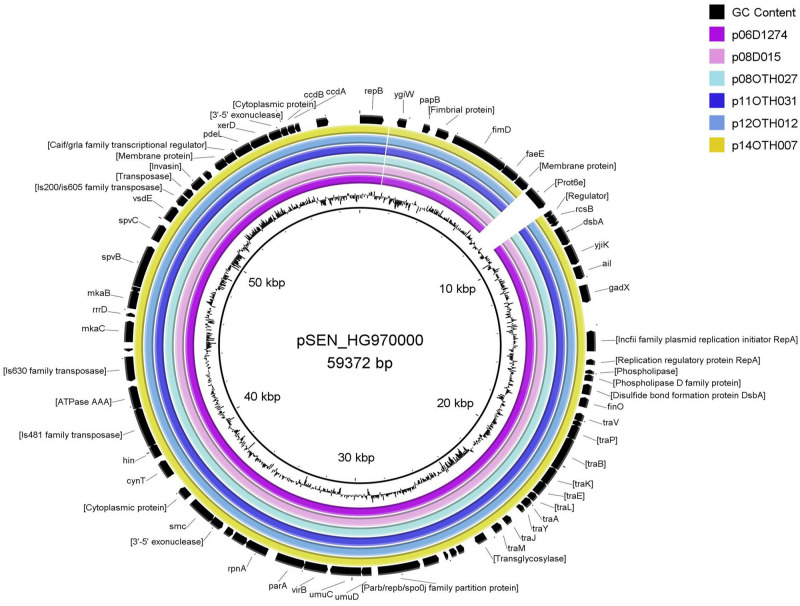
BRIG diagram comparing unusual *S*. Enteritidis plasmid sequences with that of the reference plasmid pSEN. Colored rings represent 100% similarity to the reference sequence.

**FIGURE 3:**
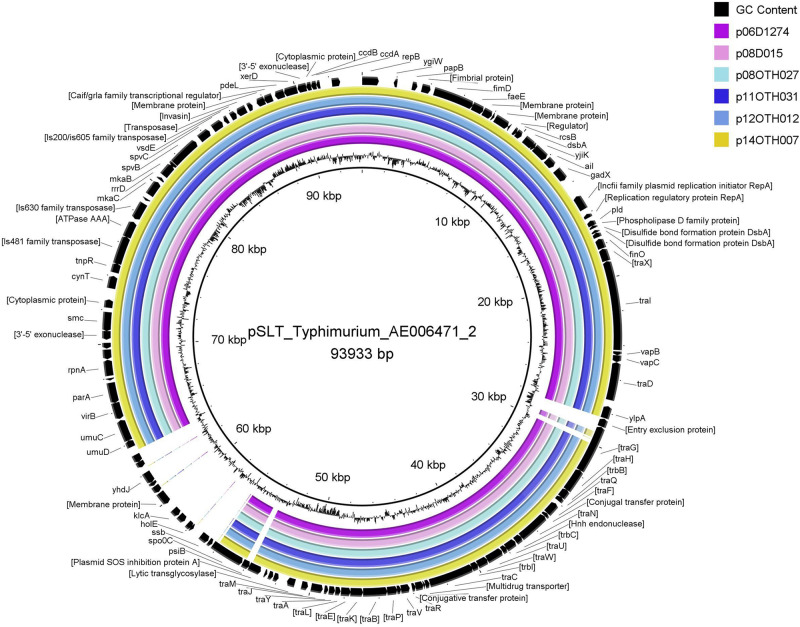
BRIG diagram comparing unusual *S*. Enteritidis plasmid sequences with that of the reference plasmid pSLT. Colored rings represent 100% similarity to the reference sequence.

The large size of these plasmids compared to the pSEN virulence plasmid carried by the P125109 reference strain of *S*. Enteritidis (59,372 bp) prompted a BLAST analysis in which high similarity of sections of these plasmids to sequence of *S.* Typhimurium reference plasmids, pSLT and pDT104, was identified, a finding consistent with the GC content (52.6%) of our plasmids compared to that of the pSEN reference (51.9%) and *S.* Typhimurium plasmids (53.1%) respectively. Direct comparison of these plasmids with pSEN, indicated a high level of similarity except for the absence of a small region around the *prot6e* gene (Figure 2). When compared with the pSLT plasmid of *S.* Typhimurium reference strain LT2 the six unusual *S*. Enteritidis plasmids were found to contain a ∼28 kb insertion with strong similarity to pSLT (Figure 3) that is not contained within pSEN. Both comparisons supported the homology of all six *S.* Enteritidis plasmids.

Full annotation of these six unusual *S.* Enteritidis plasmids, including comparison with the corresponding plasmids from the *S*. Enteritidis and *S*. Typhimurium reference strains, is provided (Supplementary Table S2). All six *S.* Enteritidis plasmids generated very similar annotation profiles with just a few differences resulting from truncation or, occasionally, complete loss of some ORFs. Notably, most feature differences between these plasmids are located within a stretch of about 2 kb in the region corresponding to positions 10–12 kb.

**FIGURE 4:**
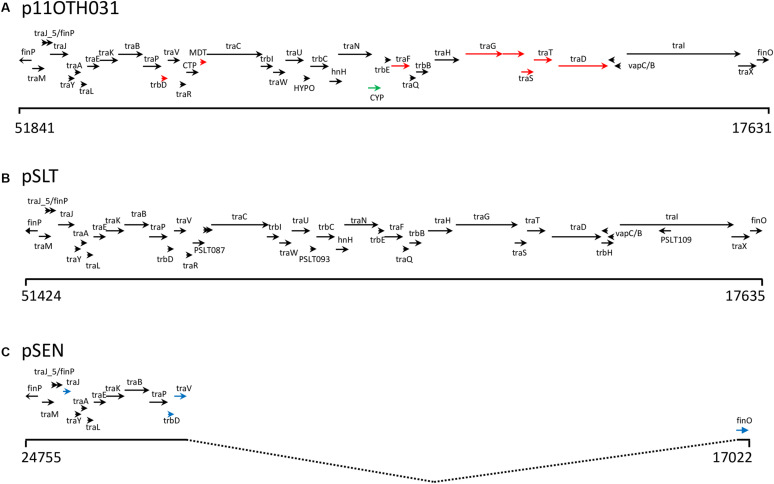
**(A)** Schematic showing the genes encoded by a 34.5 Kb region of p11OTH031 including the 28 Kb insertion harboring multiple genes of the *incF* operon. Base positions corresponding to the start and end of this region are indicated; the arrows show most of the operon transcripts running off the reverse strand. The corresponding regions of the pSLT **(B)** and pSEN **(C)** reference plasmids are also shown. Black arrows indicate similar gene lengths between all three plasmids. Red arrows indicate a different length for p11OTH031 while blue arrows indicate gene lengths specific for pSEN. The green arrow in p11OTH031 identifies a distinct gene encoding a cytosine permease not found in either pSLT or pSEN. All six *S.* Enteritidis plasmids analyzed in detail in this report exhibited very similar gene organization over this region with the following exceptions. The p06D1274 plasmid had a truncated *traI*; p08OTH027 had a truncated cytosine permease gene and a truncated *traD* gene while the other samples exhibited small variations in the length of the *traD* gene.

The 28 kb insert relative to pSEN contains a contiguous section of the F transfer (*inc*F) operon very similar to a region within the *S*. Typhimurium plasmid pSLT (Figure 4) thereby reconstituting a complete F factor transfer region comprised of 32 genes typical of this operon (Frost et al., 1994). Notably, however, all the *S.* Enteritidis plasmids contained a truncated *tra*G gene. The *FinO* gene that encodes an inhibitor of the DNA transfer process that is associated with this region was similar in length to that of the *S*. Typhimurium sequence which, depending on the true start location, encodes an ORF of either 187 (ATG initiation) or 194 (TTG initiation) amino acids compared to the shortened ORF (140 amino acids) present in the pSEN plasmid. Virtually all the ORFs encoded by the *S.* Typhimurium-like insertion in these plasmids were located on one DNA strand consistent with the functional requirement of the *inc*F operon whereas the ORFs encoded by the region corresponding to the regular *S*. Enteritidis portion of these plasmids were distributed between both DNA strands. Differences between the divergent *S.* Enteritidis plasmids over the *inc*F operon were limited but notably there was significant variation in the number of repeats of a 9-base sequence encoding QPQ near the C terminal of the t*ra*D gene product which is believed to function as a DNA transporter; this variation was the primary determinant of the number of indels observed among the plasmids (Table 1). Compared to the homologous *S.* Typhimurium sequence one additional ORF, a cytosine permease, was present in the six S. Enteritidis plasmids (Figure 4). In addition, the S. Enteritidis plasmids lack sequence corresponding to a region of the pSLT plasmid (53797-63268), which contains several genes including the *psi B* gene (Supplementary Table S2).

### Whole Genome Comparisons

Given that these unusual *S*. Enteritidis isolates were recovered from three facilities in two provinces over a period of 8 years (Supplementary Table S1), it was of interest to determine if this was the result of clonal expansion of a strain harboring this hybrid plasmid or due to multiple independent changes to the virulence plasmid. The MLVA analysis suggested these isolates were quite similar, particularly as a result of the loss of the VNTR 3 marker, a result confirmed by interrogation of the complete genomic sequences for these samples by an *in silico* PCR analysis which found that the primer sequences for this locus are indeed missing.

**FIGURE 5:**
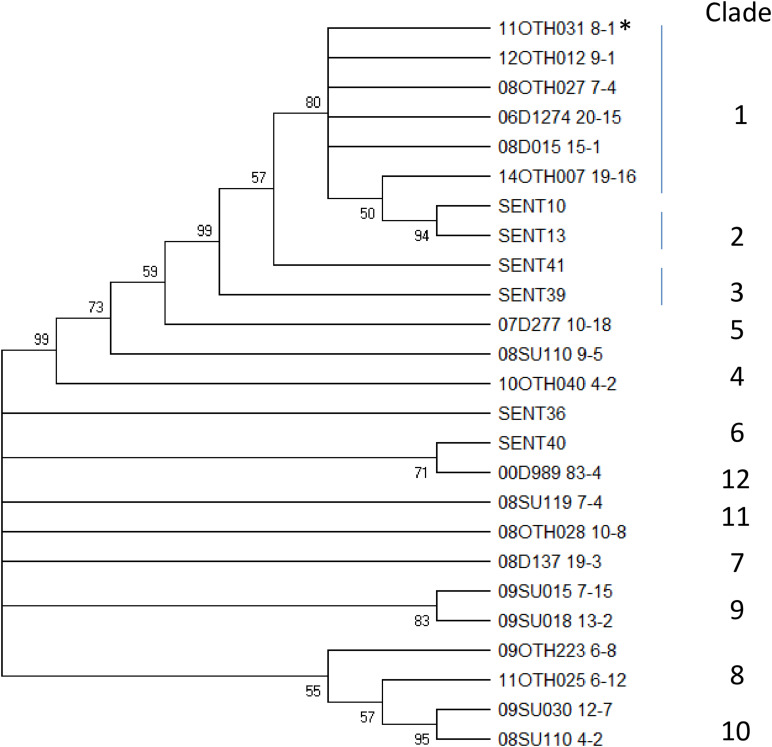
A Neighbor Joining tree illustrating the SNP typing results. This bootstrap consensus tree was generated using an alignment of 60 SNP reads for 25 *S*. Enteritidis isolates representative of 12 distinct clades including the six unusual isolates described in this report. Values at nodes indicate bootstrap values >50% and the clade designation is indicated to the right of the tree. *Indicates the isolate originally identified as clade 1.

To explore this genetic similarity further these samples were subjected to a discriminatory SNP typing protocol which scores 60 base positions located throughout the genome (Ogunremi et al., 2014b). Using this approach, the WGS data for our six sequenced samples were compared to 19 other *S.* Enteritidis sequences, several of which are included in Table 1 and which represent reference samples for many distinct groups. As shown by the number of SNPs identified in a pairwise comparison of these 25 samples (Supplementary Table S3) and the phylogeny generated using these SNPs (Figure 5), 12 distinct clades were identified. Sample 11OTH031 8-1, which had been included in that previous analysis (SENT03) and typed within clade 1, formed a reference for that clade. All five unusual *S*. Enteritidis samples recovered from Ontario facilities clustered together within clade 1 with the isolate recovered from a Manitoba facility clustering as an outlier to this group. This preliminary analysis indeed suggested that the strains harboring this unusual plasmid constitute a specific *S.* Enteritidis lineage, a conclusion further substantiated by additional studies described below.

### Prevalence of This Unusual *S*. Enteritidis Strain

To explore whether this unusual *S*. Enteritidis strain has been recovered from other samples submitted for testing to the CFIA under the umbrella of the food testing program, an in-house *Salmonella* sequence database comprising 240 *S.* Enteritidis samples was interrogated as described. This search identified a single isolate (2018-MER-0138), recovered in the province of British Columbia in western Canada in 2007 but only sequenced in 2018, which contained a plasmid with significant similarity (99.1%) to the plasmids of our six *S.* Enteritidis samples. Notably this isolate, which typed as PT9b, was again recovered from an environmental swab taken in a hatchery in which duck eggs were processed.

**FIGURE 6:**
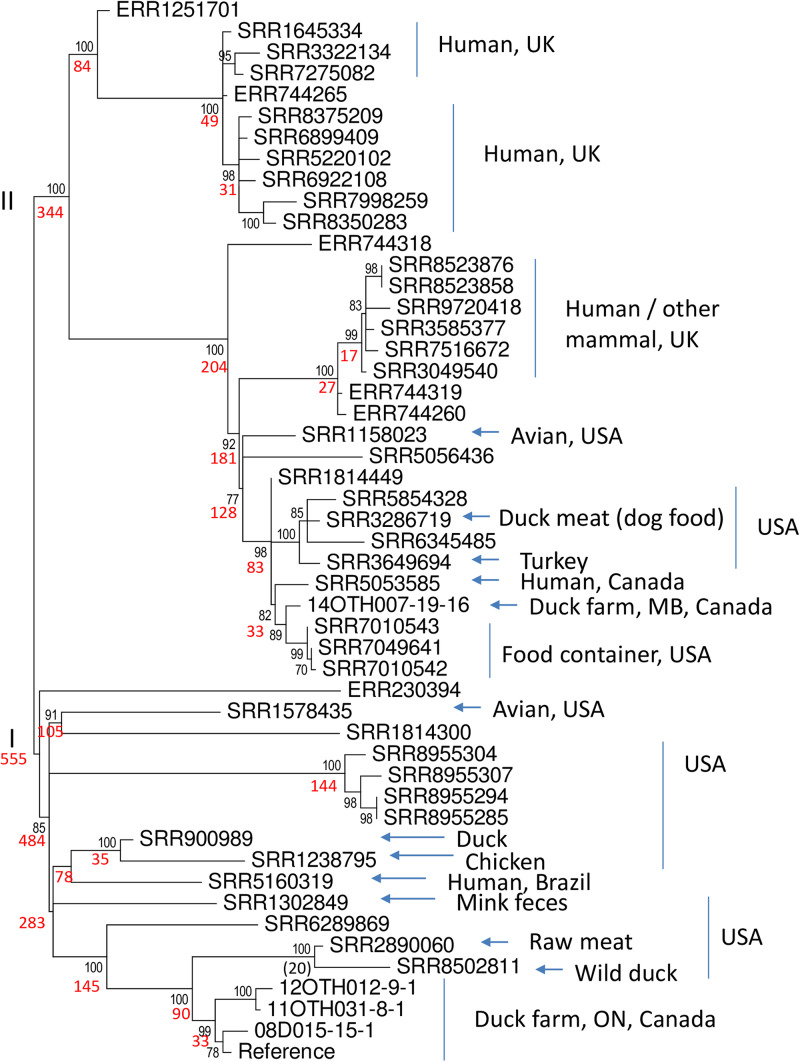
Phylogeny of 50 *S.* Enteritidis isolates. The data set includes five of the unusual Canadian samples described in this report and 45 of 54 ST-814 isolates of *S.* Enteritidis identified in the Enterobase collection as containing the unusual virulence plasmid. Using SRA data recovered from the NCBI genome database and the whole genome sequences generated in this report, a SNVphyl analysis of these whole chromosomal sequences, using 06D1274-20-15 as the reference, identified a total of 906 SNPs. These data were employed in a ML analysis, using 500 bootstrap replicates, to generate a predicted phylogeny using the General Time Reversible model of nucleotide substitution identified as the most appropriate model using modeltest. The two major clades (I and II) are identified to the left of the tree. Black numbers at nodes indicate per cent bootstrap values >70 for the clade to the right while number of SNPs within each of these clades is indicated in red. The origins of the samples with respect to sample type and country or region are summarized to the right of the tree where known. Such metadata were missing for nine samples including all six with an ERR designation and SRR1814300, SRR1814449, and SRR5056436.

To further investigate the extent to which *S*. Enteritidis isolates harboring this unusual plasmid have been recovered elsewhere, these six isolates were first subjected to *in silico* MLST analysis using the Enterobase tool and designated as ST-814. All members of this type in the Enterobase database were screened for the presence of sequence closely related to that of plasmid p08OTH027. Of just 128 samples that comprise the ST-814 group, 54 samples, as summarized in Supplementary Table S4, contained sequence having >99% identity to the reference plasmid. While all are predicted to represent *S.* Enteritidis based on their whole genome sequences it is notable that some were originally identified as other serovars, particularly *S.* Typhimurium, perhaps based on evidence gained from the unusual nature of their plasmid. SRA data for forty-five of these isolates were recovered from NCBI and these whole chromosome sequence data were used together with the MiSeq data for five of our Canadian isolates to generate a ML phylogeny based upon 906 SNPs identified by a SNVPhyl analysis (Figure 6).

Notably the tree identifies two major clades, I and II, represented by 555 and 344 SNPs respectively. Members of Clade I differed from each other by 0–156 SNPs (identity = 82.8 – 100%) whereas members of Clade II differed by 1 – 142 SNPs (identity = 84.3 – 99.9%). Statistical analysis of the distribution of SNPs in the two clades indicated that they represented distinct subgroups within the ST-814 organisms (Mann–Whitney test, two tailed, *U* = 19203, *p* < 0.05). Apart from one unannotated outlier, Clade I is comprised exclusively of samples recovered from the Americas. Included within this clade are four of the isolates recovered from Ontario duck-producing facilities which clearly form a highly conserved subgroup within a larger collection of samples from the United States. A single human isolate from Brazil also falls into this clade. The second larger clade (II) also contains several isolates from North America including the single isolate from a Manitoba duck-producing facility together with a closely related human isolate recovered in Canada. In addition, two distinct subgroups of isolates within this clade originated in the United Kingdom either from humans or other mammals. While the original sources of these human infections remain unknown, some isolates within the ST-814 group, which could not be included in the phylogeny due to their unavailability in the NCBI SRA, were obtained from both geese and ducks. The North American samples originated from a wide range of products including animal feed, several poultry species and one isolation from a mallard. The time frame of these isolations is broad ranging from 1938 (of unknown source and location) to the present with most samples recovered post 2000 (Supplementary Table S4). We thus conclude that this unusual *S.* Enteritidis plasmid is harbored by a relatively rare lineage which has over time spread over a wide geographical area.

## Discussion

While many serovars of *Salmonella enterica* subspecies *enterica* are thought to exhibit a high degree of clonality, this is especially true of *S.* Enteritidis which has emerged as an important foodborne pathogen and for which isolate subtyping is especially important in support of food outbreak investigations. Of the 63 isolates initially compared in this study (Supplementary Table S1) three had been previously sequenced and found to be very similar genetically to the reference *S.* Enteritidis strain P125109 (Ogunremi et al., 2014a). In contrast, this report has identified a rare but widely dispersed lineage of *S*. Enteritidis which carries a very unusual virulence plasmid differing from the typical plasmid carried by this serovar in two respects: loss of the *prot6e* gene and acquisition of a 28 kb sequence corresponding to a region of the *incF* operon. These findings are significant at many levels.

The clonality of the *S*. Enteritidis isolates recovered from duck-producing facilities as described in this study is strongly supported. Five of the original isolates that underwent exhaustive characterisation in this report were analyzed using a SNP-based classification tool (Ogunremi et al., 2014b) targeting 60 distinct SNPs distributed throughout the genome and were identified as belonging to the rare clade 1 type (Figure 5). These samples clustered together with an additional four isolates (08D015 13-7, 08OTH012 8-5, 08D015 19-1 and the partially characterized 12OTH019 10-1 (Supplementary Table S1) as a distinct MLVA group (Figure 1). PFGE of all 10 isolates also identified profiles distinct from the other 53 *S.* Enteritidis isolates examined by both the *BlnI* and *XbaI* restriction endonucleases. With respect to phage type all were either PT9b or a closely related atypical type. Finally, the six sequenced isolates were all assigned to MLST group ST-814, a relatively rare type of just 128 samples within >70,000 *Salmonella* isolates submitted to the Enterobase database. Thus, while only six *S.* Enteritidis isolates were fully characterized genetically in this report, the evidence suggests that ten isolates of this unusual strain were recovered.

The highly unusual nature of the virulence plasmid in this *S*. Enteritidis strain is most noteworthy due to loss of the *prot6e* gene commonly employed to identify *S.* Enteritidis and the acquisition of a 28 kb fragment of DNA carrying several genes of the *incF* operon. Again, the limited genetic variability between the plasmids harbored by all six characterized isolates (Figures 2–4) supports the clonality of the strain and its emergence and spread from a single event. The identification of the insertion sequence as being virtually identical to a portion of the *S.* Typhimurium virulence plasmid strongly suggests that the acquisition of this sequence was the result of a single horizontal transmission event, an occurrence that would certainly be facilitated by the co-circulation of these two *Salmonella* serovars in poultry environments as observed in a previous study (Nadin-Davis et al., 2019).

These significant changes identified in the virulence plasmid of these unusual *S*. Enteritidis isolates, gives them intermediate characteristics between the two commonest serovars of *Salmonella*. As part of the conjugative machinery the virulence plasmid of *S*. Typhimurium may exclude the entrance of other plasmids, a process that may help to protect the conjugative plasmid and ensure its survival by precluding lethal zygosis (Garcillán-Barcia and de la Cruz, 2008). However, the t*raG* gene is truncated in the *S*. Enteritidis plasmids and given the role of this gene in the conjugative transfer process (Firth and Skurray, 1992) this may contribute to an expected defect in the conjugative ability of the plasmid despite the acquisition of many other *tra* genes from the Typhimurium source (Figure 3). The changes observed in these Enteritidis plasmids appear to suggest an attempt of a stable but smaller Enteritidis virulence plasmid (59 kb) to revert to a larger ancestral plasmid from which those of Typhimurium and Enteritidis were derived (Rychlik et al., 2006). If the objective was to reconstitute a functional conjugative process in an Enteritidis isolate, the evidence in our study suggests that the attempt was not successful because some critical genes were “left behind.” However, the conjugative ability of these unusual plasmids has not been tested so this conclusion remains speculative. The *psiB* gene, which encodes the anti-SOS factor, is also missing from the virulence plasmid of this unusual *S*. Enteritidis strain as also observed for the pSEN reference plasmid. In its absence, the plasmid may be able to induce a stress response which can affect the chromosome or cause rearrangement of the plasmid (Baharoglu et al., 2010). We speculate that the lack of *psiB* could conceivably have played a role in the changes in the plasmids that resulted in the loss of the *prot6e* gene.

The changes identified in the virulence plasmid of this unusual *S.* Enteritidis strain may be directly related to the equally unusual chromosomal changes, a proposition which could be addressed by a comprehensive evaluation of the plasmid structure in each *S.* Enteritidis clade, but which is beyond the scope of this study. However, it is well established that *Salmonella* plasmids can alter the characteristics of chromosomally encoded features. For example, the *incX* plasmid is able to effect a phage type conversion of the organism from type 1 and 4 to 6a, and 8 to phage type 13 (Ridley et al., 1996) presumably by altering the receptors such as the O antigen, to which a phage can bind. Taken together, changes observed in both the plasmid and chromosome of this *S*. Enteritidis strain are unique at many levels. It will be interesting to evaluate the effect these changes might have on the virulence of the organism using a new AmpliSeq assay to interrogate the bacterial transcriptome.

The loss of the *prot6e* gene from this plasmid clearly confounds use of this target as a highly discriminatory marker for this serovar and suggests that other markers such as the *sdf1* locus (Nadin-Davis et al., 2019) may be a better target for *S.* Enteritidis identification. Loss of this gene may also have implications for the nature by which this serovar is able to spread given that the product of the *prot6e* gene, which is involved in fimbrial biosynthesis, is believed to contribute to *S*. Enteritidis persistence in eggs thereby facilitating the transmission of the bacterium following egg consumption (Clavijo et al., 2006). Thus, although propagation of this strain in eggs has not been investigated in these studies it is quite possible that loss of *pro6e* limits the survival of *S*. Enteritidis in eggs and thus decreases the incidence of human disease due to consumption of contaminated eggs. Notably none of the Canadian isolates harboring this plasmid were recovered directly from eggs. Apart from one human case which yielded an isolate similar to that recovered from a Manitoba hatchery (Figure 6), they were all environmental samples recovered from duck producing facilities. The additional 54 isolates of group ST-814 which were also identified as harboring this unusually large virulence plasmid originated from several sources including multiple human cases from the United Kingdom as well as meat or animal feed containing duck meat (Supplementary Table S4 and Figure 6). Given the relatively rare association of this *S.* Enteritidis strain with humans and the probable limitations of this organism to propagate efficiently in eggs we speculate that human infection with this strain is more likely to be associated with contamination of poultry meat rather than eggs, a suggestion consistent with the documented source of these isolates. Clearly, however, this strain does cause human disease and is thus a public health concern particularly given the possibility that cases might be missed due to loss of the *prot6e* diagnostic marker. In Canada, given the recovery of this *S.* Enteritidis strain from duck producing facilities in three provinces (Ontario, Manitoba, and British Columbia), it is clearly circulating over a wide geographical range in this host species and presents as either a PT9b or a closely related type. Unfortunately, apart from one PT9b sample, phage types for the remaining 53 ST-814 samples harboring this plasmid are not available so it is unknown if association with PT9b is a common feature of this unusual virulence plasmid. Interestingly, in the United Kingdom it has been reported that while PT9b *S.* Enteritidis is not often associated with human disease there has been an increased incidence of this biotype in duck producing facilities in contrast to the overall decline of *Salmonella* in poultry populations (Carrique-Mas et al., 2008). Phage typing of some of the samples of this collection would help address this issue. The single isolation of this strain from a wild mallard recovered in the US state of Indiana is notable and may indicate a means by which migrating birds could have spread this strain widely but further study on the precise source of isolates of this type is needed before such an association can be concluded.

## Data Availability Statement

The datasets generated for this study can be found in the GenBank under accession numbers GCA_008728015.1 (2006D1274-20-15), GCA_008727985.1 (2011OTH031-8-1), GCA_008727965.1 (2008D015-15-1), GCA_008727955.1 (2008OTH027-7-4), GCA_008727915.1 (2014OTH007-19-16), and GCA_008727855.1 (2012OTH012-9-1).

## Author Contributions

SN-D co-ordinated all the aspects of this work including drafting of the manuscript. LP performed all Illumina and PGM sequencing. JC and M-OD undertook the MinION sequencing and performed the all bioinformatics analysis. TB and JD maintained the culture collection and were responsible for provision of all isolates together with their source data. OA performed the MLVA. RA provided the PFGE results. DO arranged for the whole genome SNP typing analysis and contributed to the interpretation of all results. All authors contributed to the writing of the manuscript.

## Conflict of Interest

The authors declare that the research was conducted in the absence of any commercial or financial relationships that could be construed as a potential conflict of interest.

## Footnotes

1 https://github.com/rrwick/Unicycler

2 https://github.com/chmaraj/In_silico_PCR

3 https://github.com/duceppemo/bacteria_genome_assembly/blob/master/bda.py

4 https://samtools.github.io/bcftools/

5 https://enterobase.warwick.ac.uk/
